# Subthalamic nucleus stimulation and levodopa modulate cardiovascular autonomic function in Parkinson’s disease

**DOI:** 10.1038/s41598-017-07429-9

**Published:** 2017-08-01

**Authors:** Kai Li, Rocco Haase, Heinz Rüdiger, Manja Reimann, Heinz Reichmann, Martin Wolz, Tjalf Ziemssen

**Affiliations:** 1 Autonomic and neuroendocrinological lab, Center of Clinical Neuroscience, University Hospital Carl Gustav Carus, Dresden University of Technology, Fetscherstr. 74, 01307 Dresden, Germany; 2 Department of Neurology, University Hospital Carl Gustav Carus, Dresden University of Technology, Fetscherstr. 74, 01307 Dresden, Germany; 3 Department of Neurology, Beijing Hospital, National Center of Gerontology, China. No. 1 DaHua Road, Dongdan, Beijing, 100730 China

## Abstract

We aimed to explore the effects of bilateral subthalamic nucleus stimulation and levodopa on cardiovascular autonomic function in Parkinson’s disease. Twenty-six Parkinson’s disease patients with bilateral subthalamic nucleus stimulation in a stable state were tested under stimulation off and dopaminergic medication off (OFF-OFF), stimulation on and dopaminergic medication off (ON-OFF), and stimulation on and medication (levodopa) on (ON-ON) conditions by recording continuously blood pressure, ECG, and respiration at rest, during metronomic deep breathing, and head-up tilt test. Thirteen patients were diagnosed as orthostatic hypotension by head-up tilt test. Baroreflex sensitivity and spectral analyses were performed by trigonometric regressive spectral analysis. Subthalamic nucleus stimulation and levodopa had multiple influences. (1) Systolic blood pressure during tilt-up was reduced by subthalamic nucleus stimulation, and then further by levodopa. (2) Subthalamic nucleus stimulation and levodopa had different effects on sympathetic and parasympathetic regulations in Parkinson’s disease. (3) Levodopa decreased baroreflex sensitivity and RR interval only in the orthostatic hypotension group, and had opposite effects on the non-orthostatic hypotension group. These findings indicate that subthalamic nucleus stimulation and levodopa have different effects on cardiovascular autonomic function in Parkinson’s disease, which are modulated by the presence of orthostatic hypotension as well.

## Introduction

Dysfunction of the cardiovascular autonomic nervous system (ANS) is common in Parkinson’s disease (PD), and can undermine quality of life, as well as relate to a higher mortality^1, 2^. Spectral and baroreflex analysis are valuable tools for quantitatively evaluating the fluctuations of heart rate and blood pressure, and reflect the parasympathetic and sympathetic regulations of cardiovascular function^3^. Patients with PD showed impairment in heart rate variability and baroreflex sensitivity (BRS) in multiple studies^4–8^. Ischemic heart disease is a main cause of death in PD^9^, and abnormalities in spectral and baroreflex analysis are powerful predictors for increased mortality due to ischemic heart disease^10, 11^. Therefore, heart rate variability and BRS impairments may be associated with increased cardiovascular mortality in PD. Deep brain stimulation of the subthalamic nucleus (STN-DBS) and levodopa are crucial treatments for PD. Despite their widespread application, the influences of STN-DBS and levodopa on cardiovascular ANS function in PD are still to be elucidated.

Research on the effect of STN-DBS on the cardiovascular ANS function has gained divergent results so far. Some studies reported a positive influence: it could ameliorate orthostatic hypotension (OH), correct heart rate variability abnormality, and improve BRS^12–14^. In contrast, some studies found no significant effects of STN-DBS on the cardiovascular ANS in PD^15–17^. Disparities in study protocols and small sample size may be a major reason for the above disparities^12–14^.

As levodopa is the precursor of dopamine and norepinephrine, it is highly probable to affect the cardiovascular autonomic function. Some studies showed that levodopa could ameliorate heart rate variability abnormality, and did not exacerbate OH^18–22^. However, more studies demonstrated that levodopa lowered blood pressure and even enhanced OH^15, 23–28^. In addition, PD patients with and without OH may have extensive differences in autonomic dysfunction and might even be classified as two subtypes^29^. In a study by Trachani and colleagues, PD patients with and without OH seem to respond differently to STN-DBS and levodopa regarding cardiovascular autonomic function^17^.

This study aimed to investigate the influence of STN-DBS and levodopa to cardiovascular autonomic function in PD using ON-OFF comparisons in a large patient sample. Furthermore, we wanted to explore whether PD patients with and without OH responded differently to STN-DBS and levodopa.

## Patients and Methods

### Patients

Twenty-six patients with PD who received bilateral STN-DBS at least 6 months ago were enrolled. The diagnosis of PD was based on the UK Brain Bank criteria. PD medication and STN-DBS parameters were stable for at least 4 weeks prior to study inclusion. Patients with diabetes, significant cerebrovascular or cardiovascular diseases were excluded. OH was diagnosed according to the consensus proposed by Kaufmann *et al*. during head-up tilt test (HUT)^30^. The study was in accordance with relevant guidelines and regulations, and approved by the Institutional Review Board of University Hospital Carl Gustav Carus. This study was carried out according to the Declaration of Helsinki. All the subjects gave written informed consent prior to participation.

### The STN-DBS surgery

A pre-surgery MRI was conducted to plan the trajectories to the STN. We used the following coordinates on both hemispheres to determine the target points: x (lateral distance from the midline) = 12, y (anteroposterior distance from the AC) = 3, and z (height relative to the AC line) = 4. Individual adjustments were made according the T2-weighted images. Surgery was performed under local anesthesia using the ZD stereotactic system. The target points were determined during operation by microelectrode recording (ISIS MER System, Inomed) and intraoperative neurological testing of stimulation effects. Electrodes (Medtronic 3389) were implanted and leads were fixed at the burr hole. The pulse generator (Activa PC Modell 37601; Medtronic) was implanted infraclavicularly or abdominally and activated. Following operation, antiparkinsonian medications were tapered, and the STN-DBS parameters were adjusted to obtain the best clinical performance. At the time of our study, the mean (standard deviation) of the stimulation voltage, pulse width, and frequency of our patients were: 2.25(0.92) V, 61.15(5.83) μs, and 131.92 (9.71) Hz respectively.

### Autonomic function examination procedure

The cardiovascular ANS function evaluations were performed under three different conditions: OFF-OFF (at least 12 h STN-DBS off and anti-parkinsonism drugs free), ON-OFF (1 h after STN-DBS switched on, and at least 12 h off anti-parkinsonism medication), ON-ON (1 h after taking 200 mg levodopa/50 mg benserazide, and under normal STN-DBS stimulation). Medications other than antiparkinsonian drugs were taken as usual.

At each occasion, the autonomic testing was performed during the morning, and in a specialized autonomic laboratory with controlled humidity and temperature. Beat-to-beat blood pressure, heart rate and respiratory rate were monitored continuously using the SUEMPATHY device (Suess Medizin-Technik, Aue, Germany), which included the non-invasive blood pressure monitoring CBM3000 device (Nihon Colin Co., Komaki, Japan). After instrumentation, the patients rested in a supine position on a tilt table for 20 min to reach a steady state, the last 5-minute recording of this resting stage was used for further TRS analysis. Then metronomic deep breathing at 6 cycles/min for 2 min was performed. The maximum and minimum RR interval (RRI) during each breathing cycle was measured. Then the average of the maxinum/minimum RRI ratio of three consecutive breathing cycles was calculated as E/I ratio. Thereafter, the patients stayed at least for 20 min to be stable again in heart rate and blood pressure. Subsequently the subjects were tilted up to a 60° upright position within 15 s for 5 min during HUT testing. During the HUT test under ON-ON condition, OH was defined as a reduction of SBP of at least 20 mmHg or DBP of at least 10 mmHg within 3 min of tilting.

### Spectral and baroreflex analysis by trigonometric regressive spectral (TRS) analysis

The recorded data of RRI and systolic blood pressure (SBP) were processed by TRS analysis^3, 31, 32^. TRS is a newly developed and advanced analytical technique^3, 31^. The algorithm of TRS analysis provides a pure physiological spectrum using trigonometric regression. In contrast to Fast Fourier Transformation, TRS does not need interpolation on non-equidistant RRIs, and can analyze a data segments as short as 25 s^3, 31^. BRS was calculated as the slope of the regression line of coherent pairs of the detected oscillations of RRI and SBP (cross correlation coefficient >0.7)^32^. The good performance of TRS in analyzing BRS was confirmed by the EuroBavar Study^33^. Stable global data segments of 1.5–2 min were selected for analysis using local time windows of 30 seconds, and the artifacts and extrasystoles were manually identified and corrected using the TRS software. Mean RRI, SBP, the standard deviation of NN intervals (SDNN), the root mean square of successive differences (RMSSD), and the following frequency-domain parameters of the heart rate variability and SBP variability were calculated. Low frequency power of heart rate variability (RR–LF), high frequency power of heart rate variability (RR–HF), low frequency power of SBP oscillations (SBP-LF), and high frequency power of SBP oscillations (SBP-HF) were expressed as relative values which are the proportions (in percent) of LF and HF powers in the total power. LF power is the spectral band between 0.04 and 0.15 Hz, and HF power is the spectral band between 0.15 and 0.4 Hz. RR-LF reflects a mixture of sympathetic and parasympathetic activity, and SBP-LF relates to the sympathetically mediated peripheral vasomotor tone. RR-HF represents the parasympathetic cardiovagal tone, and SBP-HF results from mechanical effects of breathing on intrathoracic pressure and/or cardiac filling (independent of autonomic activity). LF/HF ratio of RRI represents the balance between sympathetic and parasympathetic tone in cardiovascular ANS^3, 34, 35^.

### Statistical analysis

All statistical analyses were performed using SPSS for Windows (Version 23.0. Armonk, NY: IBM Corp). Data are presented as mean ± standard deviation unless stated otherwise. The Kolmogorov-Smirnov test was used to evaluate data normality. Logarithmic transformation was used if applicable. Between-group comparisons of demographic and clinical characteristics were performed via t-test, Mann-Whitney U test, or Fisher’s exact test. Changes in cardiovascular ANS parameters across different STN-DBS and medication states were analyzed by repeated measures ANOVA, with OH as the between-subjects factor. The contrast was set as repeated. Greenhouse-Geisser correctional adjustment was used if the assumption of sphericity was violated.

Comparisons of the parameters between at rest and deep breathing, and between supine position and tilt-up were carried out with paired t-test. The changes (Δ values) of cardiovascular parameters from at rest to deep breathing and from supine position to tilt-up were calculated under different STN-DBS and anti-parkinsonism medication conditions, and the Δ values under the three STN-DBS and levodopa conditions were analyzed by repeated measures ANOVA or Friedman Test. All tests were two-tailed and p < 0.05 was considered as statistically significant.

## Results

### Study population

Clinical and demographical features of the patients are presented in Table 1. There were 18 males and 8 females. Mean duration from surgery to investigation was 12.35 ± 2.78 months. Thirteen of the PD patients had OH during HUT in ON-ON state, while 13 had not. Mean age of the OH group was 71.62 ± 2.50 years, which was older than the non-OH group (64.31 ± 7.73 years) (p = 0.006). Disease duration was similar between the two groups (17.37 ± 4.42 and 14.72 ± 4.64 years, respectively, and p = 0.149). Under ON-ON condition, motor impairment of the two groups was comparable (UPDRSIII 17.54 ± 6.96 and 22.92 ± 8.31, p = 0.086; Hoehn & Yahr staging 1.92 ± 0.86 and 2.12 ± 0.85, p = 0.571). Daily levodopa equivalent dose was calculated for all antiparkinsonism agents^36^. The two groups had similar daily levodopa equivalent doses (858.46 ± 212.45 and 1166.62 ± 600.95, p = 0.102).

**Table 1:** Demographic and clinical characteristics of the patients with Parkinson’s disease.

Patient number	Age (years)	Sex	BMI	Age of PD Onset (years)	Duration of PD (years)	Duration from Surgery to examination (months)	Orthostatic hypotension	Hoehn and Yahr Staging		UPDRS III			LEDD (mg)
								OFF-OFF	ON-ON	OFF-OFF	ON-OFF	ON-ON	
1	72	M	26.5	54	19	17	Y	3.0	2.0	16	11	5	733
2	74	F	24.2	57	17	18	Y	4.0	4.0	36	25	25	600
3	71	F	23.9	51	20	14	N	4.0	3.0	44	30	18	810
4	68	M	21.0	48	21	14	N	5.0	3.0	42	37	29	1098
5	69	M	27.8	52	18	13	N	4.0	2.0	42	23	16	300
6	54	F	28.6	38	16	12	N	3.0	1.0	51	32	16	1380
7	63	F	22.5	46	17	13	N	2.5	2.0	43	27	21	625
8	71	M	24.6	61	11	18	N	4.0	3.0	36	26	19	1171
9	74	F	31.4	62	13	13	Y	2.5	1.5	26	18	13	1250
10	75	M	25.0	57	19	14	Y	3.0	2.5	48	28	20	903
11	67	M	22.1	55	12	15	Y	4.0	2.5	37	32	27	860
12	76	M	24.0	55	20	14	N	5.0	2.5	67	54	34	1624
13	71	M	23.0	55	16	13	Y	1.5	1.0	26	16	14	1000
14	72	M	30.4	55	17	13	Y	4.0	2.5	45	35	26	700
15	68	M	28.1	41	27	11	Y	2.5	1.0	33	19	11	830
16	62	M	25.2	55	7	11	N	5.0	2.5	56	30	25	1384
17	69	F	23.0	54	14	10	Y	1.5	1.0	37	25	20	1035
18	62	F	24.0	49	13	10	N	4.0	1.0	76	32	21	800
19	65	M	42.0	54	11	10	N	5.0	3.0	58	39	32	1815
20	56	M	22.0	42	14	10	N	1.5	1.0	25	24	19	2129
21	72	M	29.6	61	12	9	Y	2.5	2.0	40	29	24	865
22	70	M	23.7	53	17	7	N	4.0	2.5	48	41	40	1830
23	73	M	22.0	57	17	10	Y	4.0	2.0	48	25	17	1159
24	70	M	24.6	47	23	12	Y	3.0	2.0	38	27	19	700
25	74	M	23.2	54	21	11	Y	2.0	1.0	39	10	9	525
26	49	F	24.7	43	7	10	N	1.5	1.0	20	14	11	200

BMI = body mass index, F = female, M = male, LEDD = levodopa equivalent daily dose, PD = Parkinson’s disease, OFF-OFF = both off STN-DBS and off dopaminergic medication, ON-OFF = on STN-DBS and off dopaminergic medication, ON-ON = both on STN-DBS and on levodopa, UPDRS = Unified Parkinson’s Disease Rating Scale.

### Influence of levodopa and STN-DBS on cardiovascular autonomic function at rest

**Figure 1:**
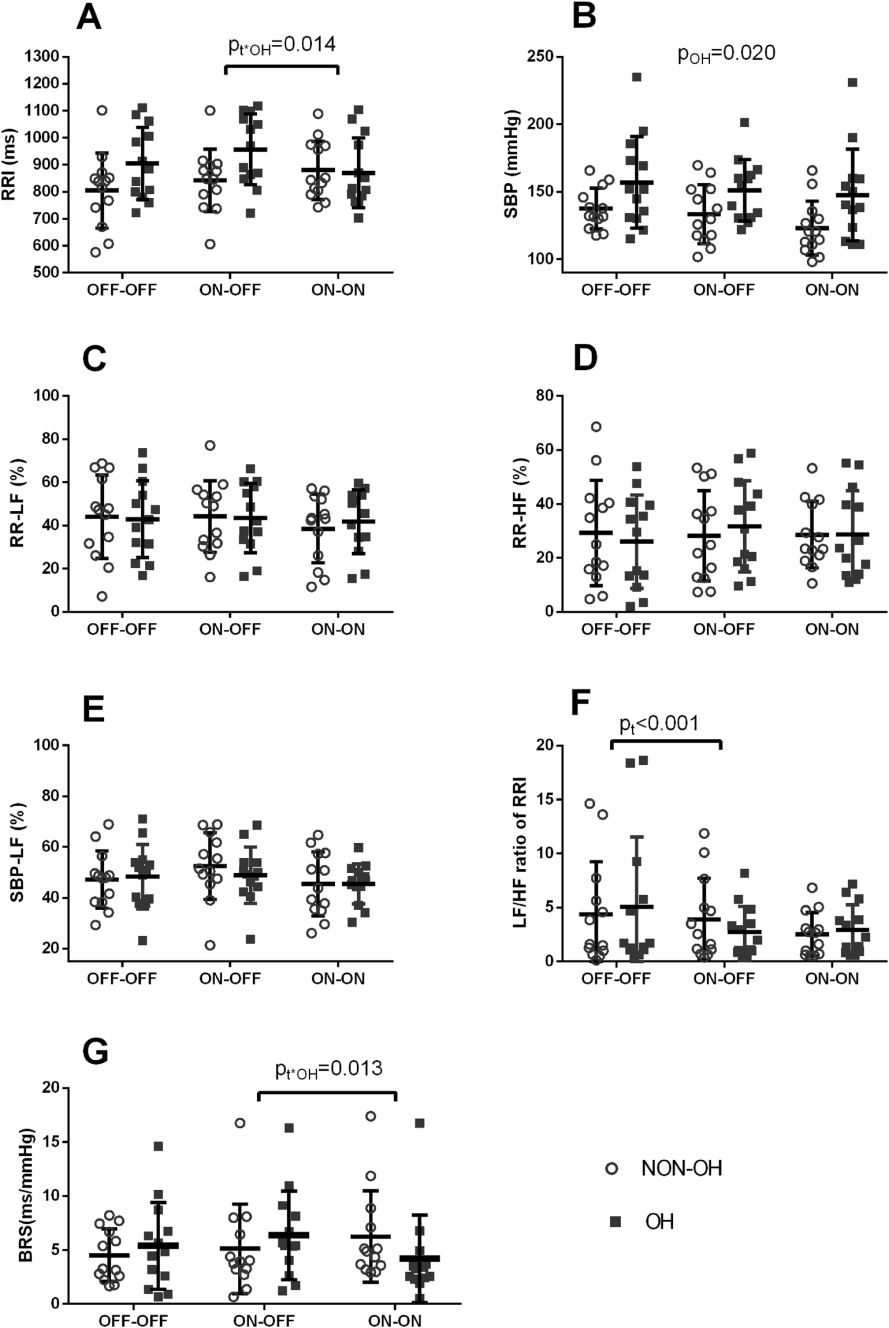
Cardiovascular autonomic function parameters at rest. p_t_ is for repeated measures, p_OH_ for the effect of OH, and p_t*OH_ is for an interaction between repeated measures and OH. BRS = baroreflex sensitivity, HF = high frequency, LF = low frequency, OH = orthostatic hypotension, RRI = RR interval, SBP = systolic blood pressure; OFF-OFF = both off STN-DBS and off dopaminergic medication, ON-OFF = on STN-DBS and off dopaminergic medication, ON-ON = both on STN-DBS and on levodopa. Data are presented as the mean ± SD, and analyzed by repeated measures ANOVA.

During rest, RRI and time domain parameters were not significantly affected by STN-DBS or levodopa, but there was a trend for SBP to decrease from OFF-OFF to ON-OFF (by STN-DBS), and then further to ON-ON state (by levodopa) (p = 0.063). LF/HF ratio of RRI at rest was significantly reduced by STN-DBS, from 4.71 ± 5.64 under OFF-OFF condition, to 3.31 ± 3.17 and 2.74 ± 2.14 under ON-OFF and ON-ON conditions, (F (1.34, 32.25) = 45.97, p < 0.001). BRS and other parameters of spectral analysis were not changed by application of STN-DBS or levodopa (Fig. 1, Supplemental Table S1).

### Influence of levodopa and STN-DBS on autonomic function during metronomic deep breathing

Under OFF-OFF state, deep breathing induced a low frequency power predominance, which exhibited by an increased RR-LF, LF/HF ratio of RRI, and SBP-LF, as well as decreased RR-HF (for RR-LF, LF/HF ratio of RRI and SBP-LF, p < 0.001, for RR-HF, p = 0.002).

**Figure 2:**
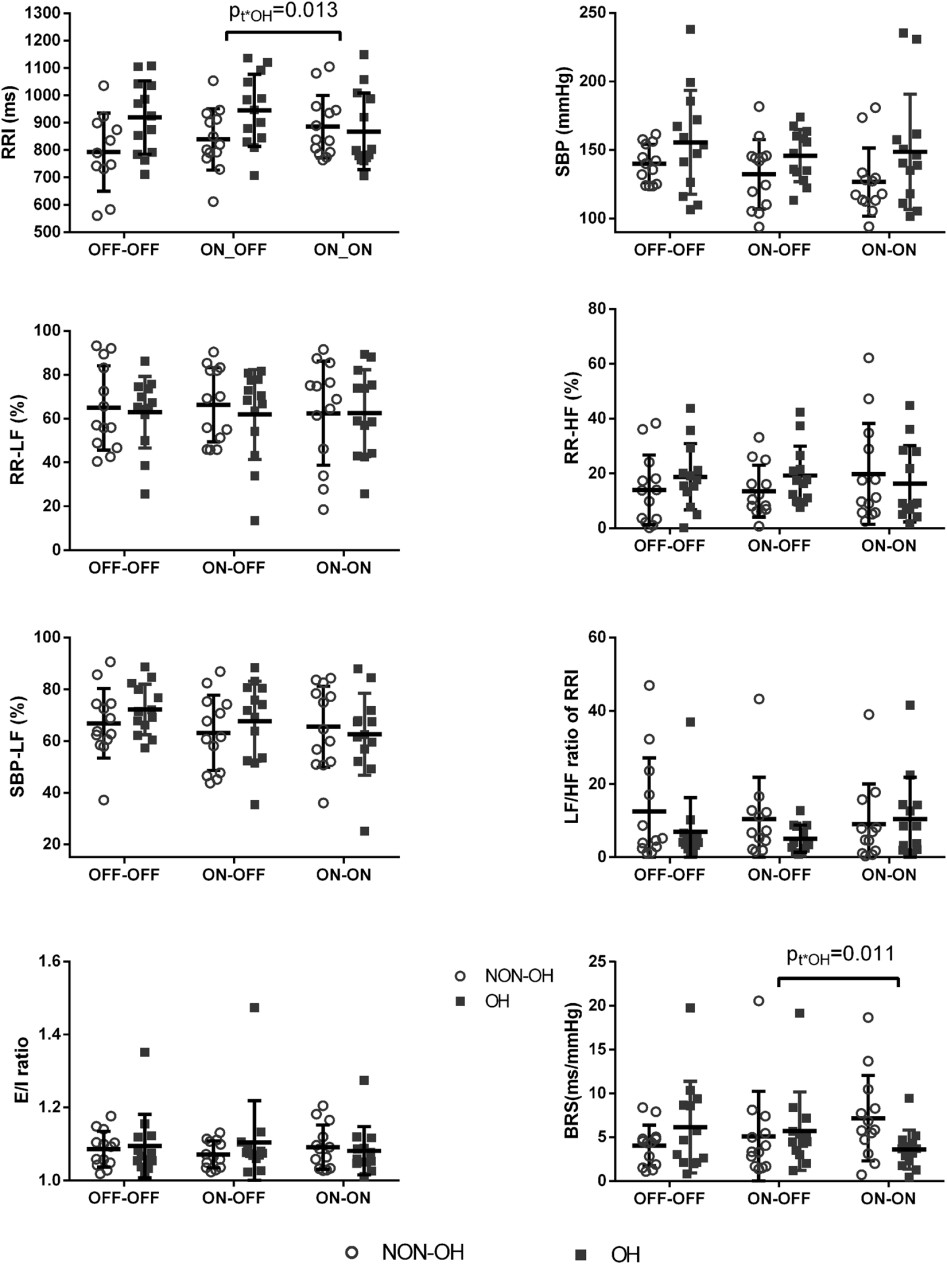
Cardiovascular autonomic function parameters during deep breathing. p_t*OH_ is for an interaction between repeated measures and OH. BRS = baroreflex sensitivity, E/I ratio = E/I ratio = expiratory/inspiratory ratio, HF = high frequency, LF = low frequency, OH = orthostatic hypotension, RRI = RR interval, SBP = systolic blood pressure; OFF-OFF = both off STN-DBS and off dopaminergic medication, ON-OFF = on STN-DBS and off dopaminergic medication, ON-ON = both on STN-DBS and on levodopa. Data are presented as the mean ± SD, and analyzed by repeated measures ANOVA.

After switching on STN-DBS and taking levodopa, the above findings comparing the parameters during deep breathing with those at rest remained the same. Comparing these parameters during deep breathing from OFF-OFF, to ON-OFF and then ON-ON, no main effect of STN-DBS or levodopa was found in RRI, SDNN, RMSSD, E/I ratio, SBP, RR-LF, RR-HF, LF/HF ratio of RRI, SBP-LF, or BRS (Fig. 2, Supplemental Table S1).

### Influence of levodopa and STN-DBS on autonomic function during HUT

**Figure 3:**
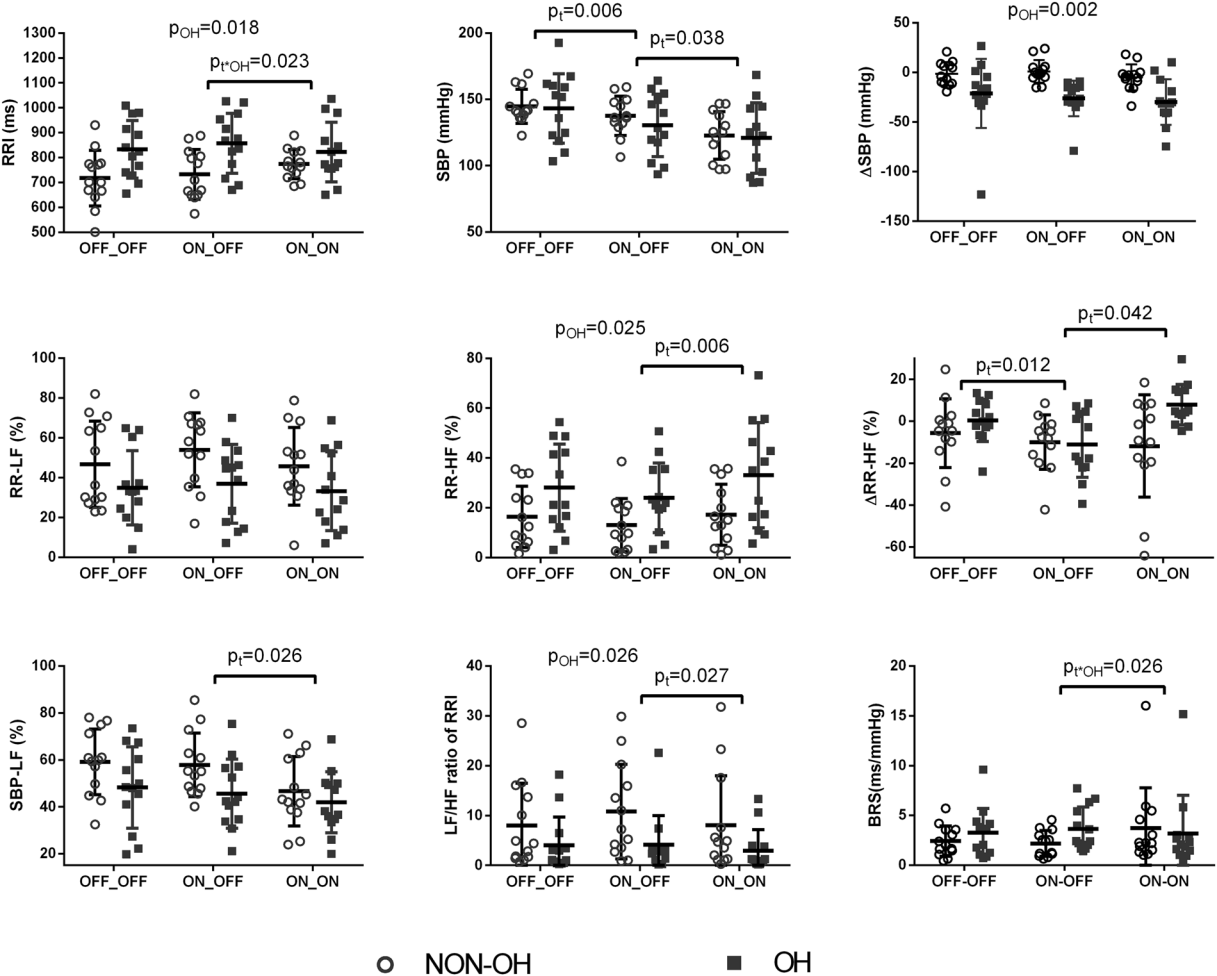
Cardiovascular autonomic function parameters during tilt-up. p_t_ is for repeated measures, p_OH_ for the effect of OH, p_t*OH_ for an interaction between repeated measures and OH. BRS = baroreflex sensitivity, HF = high frequency, LF = low frequency, OH = orthostatic hypotension, RRI = RR interval, SBP = systolic blood pressure; OFF-OFF = both off STN-DBS and off dopaminergic medication, ON-OFF = on STN-DBS and off dopaminergic medication, ON-ON = both on STN-DBS and on levodopa. Data are presented as the mean ± SD, and analyzed by repeated measures ANOVA.

As expected based on previous studies^7^, orthostasis induced a decrease in RRI, SDNN, RMSSD, SBP, and BRS, while RR-LF and SBP-LF did not change from pre-tilt to tilt-up state. The above responses to orthostasis was consistent from OFF-OFF, to ON-OFF, and ON-ON states (for RRI and BRS, all p < 0.005, for SDNN, RMSSD and SBP, all p < 0.05). However, only under ON-OFF condition, RR-HF during tilt-up was significantly lower than the pre-tilt stage (p = 0.001); while RR-HF during tilt-up was similar to that in pre-tilt phase under OFF-OFF and ON-ON conditions. Accordingly, analyzing the difference of RR-HF between pre-tilt and tilt-up states (ΔRR-HF) showed that STN-DBS led to a reduction of RR-HF from the pre-tilt to tilt-up state, and levodopa offset this reduction (F(2, 48) = 3.275, p = 0.046) (Fig. 3). Comparisons of LF/HF ratio of RRI between pre-tilt and tilt-up states obtained a result consistent with RR-HF change.

Comparing the cardiovascular ANS parameters during tilt-up under OFF-OFF, ON-OFF, and ON-ON conditions, RRI, RR-LF, and BRS were not affected by STN-DBS or levodopa; SBP, RR-HF, LF/HF ratio of RRI, and SBP-LF was influenced by STN-DBS and/or levodopa.

SBP during tilt-up was reduced by STN-DBS, and then further by levodopa. Mean SBP during tilt-up under OFF-OFF, ON-OFF, and ON-ON conditions were 144.01 ± 20.16, 134.06 ± 19.65, and 121.80 ± 22.21 mmHg, respectively (F(1.55, 37.21) = 10.46, p = 0.001) (Fig. 3, Supplemental Table S1). However, the orthostatic BP change was not significantly affected by STN-DBS or levodopa, ΔSBP during orthostasis across OFF-OFF, ON-OFF, and ON-ON states were −11.19 ± 27.51, −12.51 ± 20.30, and −17.35 ± 22.30 mmHg respectively (F(2, 48) = 1.54, p = 0.22) (Fig. 3).

RR-HF during tilt-up was increased after taking levodopa (18.53 ± 13.41% and 25.08 ± 18.77% for ON-OFF and ON-ON respectively; F (2, 48) = 4.76, p = 0.013), and this change could be explained by the aforementioned effect of levodopa on ΔRR-HF during orthostasis. LF/HF ratio of RRI showed an alteration by levodopa resulted from the change of RR-HF. SBP-LF during tilt-up was lowered by levodopa (51.72 ± 15.19% and 44.30 ± 13.88% for ON-OFF and ON-ON respectively; F(2,48) = 6.64, p = 0.003) (Fig. 3, Supplemental Table S1).

### Difference in response between the OH group and the non-OH group

The presence of OH had significant impact on the cardiovascular ANS parameters: Of course, the drop of SBP from pre-tilt to tilt-up was larger in the OH group (F (1, 24) = 12.54, p = 0.002) (Fig. 3). SBP at rest was higher in the OH group than that in the non-OH group (F (1, 24) = 6.17, p = 0.020) (Fig. 1). RRI during tilt-up in the OH group was longer (heart rate was slower) than that in the non-OH group (F (1, 24) = 6.50, p = 0.018). RR-HF during tilt-up was higher in the patients with OH than the patients without (F(1, 24) = 5.72, p = 0.025); correspondingly, LF/HF ratio of RRI during tilt-up was lower in patients with OH than patients without OH (F (1, 24) = 5.67, p = 0.026) (Fig. 3) (Supplemental Table S2).

Furthermore, the presence of OH had significant impact on the effects of levodopa on autonomic function: after taking levodopa (from ON-OFF to ON-ON), BRS increased in patients without OH, but decreased in patients with OH, and this change was similar across different states including at rest (F (2, 48) = 4.38, p = 0.018), deep breathing (F (1.39, 33.46) = 6.86, p = 0.007), and during tilt-up (F (1.62, 38.85) = 3.43, p = 0.052). Also after taking levodopa, RRI increased in patients without OH, but decreased in patients with OH, this trend was similar across at rest (F(1.40, 33.70) = 5.58, p = 0.015), deep breathing (F(1.49, 35.74) = 7.12, p = 0.005), and during tilt-up (F(1.31, 31.44) = 3.63, p = 0.056) (Figs 1, 2 and 3, Supplemental Table S2). Similarly, levodopa increased RMSSD at rest in the non-OH group, but decreased RMSSD in the OH group (F(1.373, 48) = 3.743, p = 0.049) (Supplemental Table S2).

## Discussion

In the present study, we mainly have the following 3 findings. Firstly, both STN-DBS and levodopa had a SBP lowering effect, especially during tilt-up. Secondly, spectral analysis showed that both STN-DBS and levodopa influenced the balance of parasympathetic and sympathetic tone in the cardiovascular system. Thirdly, PD patients with and without OH had a series of differences in cardiovascular ANS parameters, and the cardiovascular ANS of the two groups responded differently to levodopa. These findings can provide help in deciding therapy in PD, especially in the advanced stage.

There were three main effects of STN-DBS, STN-DBS decreased LF/HF ratio of RRI at rest, lowered SBP during tilt-up, and mediate a RR-HF reduction effect during orthostasis. Additionally, SBP at rest tended to be lower under ON-OFF than OFF-OFF state.

LF/HF ratio of RRI was reduced by STN-DBS in the present study. This parameter represents the balance between sympathetic and parasympathetic tone of cardiovascular ANS. Therefore, our findings indicate that STN-DBS attenuates cardiovascular sympathetic tone. In the study by Sverrisdóttir *et al*., STN-DBS reduced muscle sympathetic nerve activity^14^, this sympathetic attenuation effect is consistent with our findings.

The results of previous studies on the influence of STN-DBS on orthostatic adaptation varied. In two studies, STN-DBS had no effect on supine SBP^12, 15^. One of the above two studies showed no effect of STN-DBS on orthostatic SBP change and the other found a decreased orthostatic SBP drop^12, 15^. In contrast, Sverrisdóttir and colleagues revealed that stimulation of the dorsal most part of STN reduced both supine SBP and orthostatic SBP drop^14^. Our findings confirmed that at least STN-DBS did not exacerbate OH in PD, although it had some SBP lowering effect before and during tilt-up. Besides, our previous studies showed that RR-HF of healthy subjects decreased during orthostasis, which indicated a reduced parasympathetic tone to maintain blood pressure during tilt-up. However, in patients with extrapyramidal disorders, this orthostatic adaptation of RR-HF disappeared^7^. The present study showed that STN-DBS restored this parasympathetic regulation in PD, which implied a beneficial impact on orthostatic regulation.

Electrical stimulation of STN may influence cardiovascular ANS by two mechanisms. Firstly, STN has abundant connections with the structures involved in limbic system which regulates the ANS, such as ventral pallidum, thalamus, the zona incerta, and hypothalamus. In particular, STN regulate frontal cortical-basal ganglia circuits, which included a limbic circuit influencing ANS function^37–39^. Secondly, STN is close to hypothalamus and the zona incerta, electronical current may be distributed to these surrounding structures regulating ANS^13^.

Some studies reported that levodopa does not affect blood pressure or even ameliorate OH^18, 19, 21^. However, more researches showed that levodopa reduced blood pressure in supine and upright positions^15, 23–28^, and even enhanced orthostatic blood pressure fall in PD^15, 25^. When we pay attention to the details of the studies, we can find that the studies reporting no effects on blood pressure used a smaller dose. In the study showing no effect by Mehagnoul-Schipper and colleagues, the patients with PD took 100 mg levodopa/25 mg benserazide before autonomic examination^21^. Those reporting the blood pressure lowering effect of levodopa usually used higher doses. For instance, two of these studies used 200 mg levodopa/50 mg benserazide before examinations^23, 24^. Moreover, levodopa exacerbated orthostatic SBP drop at further higher doses^15, 25^. In our study, we used a dosage of 200 mg levodopa/50 mg benserazide. Therefore, our results confirm the blood pressure lowering effect of levodopa, and this effect is dose-dependent. This finding implies that in PD patients with OH, large doses of levodopa should be used with caution.

RR-HF during tilt-up was increased, and LF/HF ratio of RRI and SBP-LF during tilt-up were decreased after taking levodopa in the present study. Combining the SBP lowering effect of levodopa during tilt-up, we can infer that levodopa interfered with the sympathetic-parasympathetic regulation during orthostasis. This is consistent with previous studies. Noack *et al*. reported that after oral administration of levodopa, mean blood pressure, cardiac stroke volume, and cardiac contractility were all reduced^24^. In addition to its effect on the heart, levodopa could decrease vascular tone in the lower limbs in another study^28^.

Levodopa may influence the cardiovascular ANS through the central or peripheral pathways. It is reported that a centrally active D1 agonist could reduce blood pressure and norepinephrine, which mirrored what has been found in patients taking levodopa^26, 28, 40^. In addition, levodopa itself can directly act on the cardiovascular centers in the brainstem^27, 41^. Peripherally, dopamine is increased after the intake of levodopa, and dopamine can activate peripheral presynaptic D2 receptors, thus inhibits the release of norepinephrine from sympathetic fibers^26, 42, 43^.

It is recognized that PD patients with and without OH have a series of differences: PD patients with OH tend to be older, accompany more symptoms of other autonomic systems, have more severe impairment in autonomic structures in the brain and sympathetic nerve in the heart, and have lower plasma norepinephrine than PD patients without OH^29^. Deficiencies in the sympathetic and parasympathetic branches of the autonomic system, as well as baroreflex deficit may underlie OH in PD^29, 44, 45^. Our findings confirm that PD patients with OH have several deficiencies in coping with orthostasis, and are more sensitive to the cardiovascular adverse effect of levodopa.

Baroreflex is a vital mechanism that helps to maintain a stable blood pressure through rapid feedbacks^3^. In our previous studies, BRS was lower in patients with PD than in heathy controls^6, 7^. The present study further demonstrated that levodopa exacerbated the BRS impairment in PD patients with OH. Goldstein *et al*. have found that PD patients with OH had a lower BRS compared with those without OH, but the PD patients did not stop their antiparkinsonism medication before autonomic testing^44, 46^. In future studies on BRS in PD patients, we need to take the effect of levodopa into consideration. In addition, RRI and RMSSD also presented a similar interaction in the current study. As far as we know, our study is the first reporting different effect of levodopa on the three parameters in PD patients with and without OH. Levodopa may influence the autonomic system through several pathways, and PD patients with OH have widespread pathological changes in the autonomic system^2, 47, 48^. Based on our current knowledge, it is difficult to draw a theory explaining the effect of OH on levodopa’s influence on the cardiovascular function in PD. Therefore, the interactions between OH and levodopa need further exploration in the future.

Although our study amended several shortcomings of previous similar studies, there are several limitations in the present study. Firstly, we performed cardiovascular ANS tests under levodopa off with STN-DBS off and on, then levodopa on and STN-DBS on. If there would have been a levodopa on and STN-DBS off state, we could have better evaluated the effect of levodopa (exclude the effect of STN-DBS), but this approach would have brought more burden to the patients. Secondly, the sample size was not very large. PD patients with STN-DBS were in the advanced stage, and the procedure was complex. It is difficult to collect a very large sample. In fact, this is a less than large sample compared to previous similar studies, most of which recruited 10–20 PD patients.

In summary, STN-DBS and levodopa have distinct effects on cardiovascular autonomic function in PD. Furthermore, cardiovascular autonomic function of PD patients with and without OH responds differently to levodopa. The interaction between OH and levodopa needs to be considered in future studies on the effect of levodopa and the characteristics of cardiovascular ANS function in PD patients with OH. The underlying mechanisms warrant further investigation.

## Electronic supplementary material

Supplementary material
