# Supplementary material for: Subthalamic nucleus stimulation and levodopa modulate cardiovascular autonomic function in Parkinson’s disease
Source: Sci Rep. 2017 Aug 1;7:7012. doi: 10.1038/s41598-017-07429-9 (PMC5539113; doi:10.1038/s41598-017-07429-9)
Supplement: Supplementary file 1 — Supplementary material [file 41598_2017_7429_MOESM1_ESM.doc]

**Subthalamic nucleus stimulation and levodopa modulate cardiovascular autonomic function in Parkinson’s disease**

Kai Li, Rocco Haase, Heinz Rüdiger, Manja Reimann,

Heinz Reichmann, Martin Wolz, Tjalf Ziemssen

**Supplementary Table S1** Cardiovascular autonomic parameters of the Parkinson’s disease patients

|  | OFF-OFF | ON-OFF | ON-ON |
| --- | --- | --- | --- |
| RRI at rest (ms) | 854.62±142.22 | 899.47±134.48 | 875.37±116.73 |
| RRI during deep breathing (ms) | 858.50±143.62 | 892.52±131.45 | 877.12±125.07 |
| RRI during tilt-up (ms) | 775.23±125.75 | 795.09±125.84 | 798.19±95.75 |
| SBP at rest (mmHg) | 147.23±27.59 | 142.23±23.59 | 135.39±30.03 |
| SBP during deep breathing (mmHg) | 147.82±29.08 | 139.08±22.99 | 137.70±35.68 |
| SBP tilt-up (mmHg) | 144.01±20.16 | 134.06±19.65 | 121.80±22.21 |
| E/I ratio | 1.09±0.07 | 1.09±0.09 | 1.09±0.06 |
| SDNN at rest | 26.26±16.79 | 30.32±21.38 | 23.86±11.91 |
| SDNN during deep breathing | 25.74±18.73 | 27.36±21.15 | 24.57±17.23 |
| SDNN tilt-up | 20.25±12.75 | 21.91±10.86 | 19.32±14.75 |
| RMSSD at rest | 21.01±17.88 | 27.07±24.87 | 20.16±16.64 |
| RMSSD during deep breathing | 23.45±26.84 | 23.34±23.10 | 22.31±24.78 |
| RMSSD tilt-up | 16.58±19.37 | 14.72±12.88 | 15.48±18.65 |
| RR-LF at rest (%) | 43.55±18.24 | 43.86±16.07 | 40.20±15.12 |
| RR-LF during deep breathing (%) | 63.89±17.49 | 64.09±18.58 | 62.49±21.42 |
| RR-LF during tilt-up (%) | 40.81±20.66 | 45.48±20.73 | 39.50±20.30 |
| RR-HF at rest (%) | 27.65±18.15 | 29.95±16.59 | 28.60±14.10 |
| RR-HF during deep breathing (%) | 16.36±12.39 | 16.38±10.32 | 18.02±16.11 |
| RR-HF during tilt-up (%) | 22.22±15.99 | 18.53±13.41 | 25.08±18.77 |
| LF/HF ratio of RRI at rest | 4.71±5.64 | 3.31±3.17 | 2.74±2.14 |
| LF/HF ratio of RRI during deep breathing | 9.61±12.27 | 7.63±8.62 | 9.75±11.02 |
| LF/HF ratio of RRI during tilt-up | 6.02±7.36 | 7.48±8.45 | 5.52±7.89 |
| SBP-LF at rest (%) | 47.80±11.79 | 50.72±12.05 | 45.46±10.34 |
| SBP-LF during deep breathing (%) | 69.57±11.88 | 65.45±14.93 | 64.10±15.56 |
| SBP-LF during tilt-up (%) | 53.70±16.41 | 51.72±15.19 | 44.30±13.88 |
| BRS at rest (ms/mmHg) | 4.95±3.29 | 5.74±4.09 | 5.21±4.20 |
| BRS during deep breathing (ms/mmHg) | 5.10±4.11 | 5.40±4.73 | 5.39±4.13 |
| BRS during tilt-up (ms/mmHg) | 2.84±2.05 | 2.92±1.01 | 3.46±3.87 |

BRS=baroreflex sensitivity, HF=high frequency, LF=low frequency, RRI=RR interval, SBP=systolic blood pressure; OFF-OFF=both off STN-DBS and off dopaminergic medication, ON-OFF=on STN-DBS and off dopaminergic medication, ON-ON=both on STN-DBS and on levodopa, PD=Parkinson's disease. Data are presented as the mean ± SD.

**Supplementary Table S2** Cardiovascular autonomic parameters of the Parkinson’s disease patients, divided by whether having orthostatic hypotension (supplemental material)

|  | PD patients with OH | | | PD patients without OH | | |
| --- | --- | --- | --- | --- | --- | --- |
|  | OFF-OFF | ON-OFF | ON-ON | OFF-OFF | ON-OFF | ON-ON |
| RRI at rest (ms) | 904.45±132.89 | 956.56±130.87 | 870.66±129.25 | 804.79±138.20 | 842.37±116.14 | 880.09±107.87 |
| RRI during deep breathing (ms) | 919.38±133.86 | 945.40±131.75 | 868.64±139.60 | 797.63±130.48 | 839.63±112.17 | 885.59±113.76 |
| RRI during tilt-up (ms) | 833.09±115.10 | 857.45±120.47 | 822.35±119.87 | 717.38±111.56 | 732.74±100.28 | 774.04±58.88 |
| SBP at rest (mmHg) | 156.97±33.89 | 151.02±22.78 | 147.68±33.94 | 137.48±15.21 | 133.43±21.74 | 122.71±20.00 |
| SBP during deep breathing (mmHg) | 155.56±37.89 | 145.81±19.01 | 148.70±42.09 | 140.07±14.00 | 132.34±25.32 | 126.71±24.87 |
| SBP during tilt-up (mmHg) | 143.21±26.09 | 130.40±23.62 | 120.90±26.53 | 144.81±12.83 | 137.71±14.75 | 122.71±17.96 |
| E/I ratio | 1.09±0.02 | 1.10±0.03 | 1.08±0.02 | 1.09±0.01 | 1.07±0.01 | 1.09±0.02 |
| SDNN at rest | 30.56±19.61 | 32.98±24.47 | 22.07±12.95 | 21.96±12.77 | 27.66±18.37 | 25.64±11.00 |
| SDNN during deep breathing | 29.26±23.11 | 31.60±27.36 | 23.46±17.25 | 22.22±13.04 | 23.12±12.04 | 25.68±17.83 |
| SDNN tilt-up | 22.35±14.64 | 22.68±10.33 | 19.18±17.85 | 18.15±10.71 | 21.13±11.74 | 19.45±11.60 |
| RMSSD at rest | 22.69±18.91 | 34.31±31.72 | 16.79±13.82 | 19.33±17.38 | 19.83±12.98 | 23.53±19.01 |
| RMSSD during deep breathing | 31.29±35.60 | 28.83±27.38 | 20.50±22.48 | 15.60±9.97 | 17.85±17.24 | 24.12±27.70 |
| RMSSD tilt-up | 21.74±23.74 | 18.75±14.89 | 17.93±21.29 | 11.43±12.67 | 10.68±9.42 | 13.02±16.07 |
| RR-LF at rest (%) | 42.99±17.87 | 43.53±16.13 | 41.82±14.79 | 44,12±19,32 | 44,20±16,67 | 38.58±15.88 |
| RR-LF during deep breathing (%) | 62.91±16.30 | 61.87±20.50 | 62.52±19.80 | 64.87±19.23 | 66.31±16.97 | 62.48±23.75 |
| RR-LF during tilt-up (%) | 34.92±18.59 | 36.97±19.78 | 33.25±19.80 | 46.71±21.64 | 53.98±18.64 | 45.77±19.54 |
| RR-HF at rest (%) | 26.01±17.33 | 31.74±16.90 | 28.66±16.23 | 29.29±19.50 | 28.17±16.76 | 28.54±12.27 |
| RR-HF during deep breathing (%) | 18.75±12.05 | 19.27±10.70 | 16.26±13.94 | 13.98±12.73 | 13.50±9.46 | 19.78±18.43 |
| RR-HF during tilt-up (%) | 28.09±17.49 | 24.02±13.98 | 33.01±21.13 | 16.34±12.33 | 13.03±10.67 | 17.16±12.29 |
| LF/HF ratio of RRI at rest | 5.07±1.80 | 2.70±0.66 | 2.94±0.65 | 4.35±1.36 | 3.91±1.05 | 2.53±0.56 |
| LF/HF ratio of RRI during deep breathing | 6.94±9.34 | 5.03±3.72 | 10.42±11.45 | 12.50±14.70 | 10.44±11.42 | 9.03±10.99 |
| LF/HF ratio of RRI during tilt-up | 4.03±1.57 | 4.14±1.62 | 2.97±1.17 | 8.00±2.36 | 10.81±2.64 | 8.07±2.75 |
| SBP-LF at rest (%) | 48.35±12.69 | 48.95±11.10 | 45.48±7.92 | 47.24±11.30 | 52.48±13.13 | 45.44±12.65 |
| SBP-LF during deep breathing (%) | 72.25±9.76 | 67.75±15.52 | 62.64±15.90 | 66.89±13.53 | 63.15±14.56 | 65.56±15.71 |
| SBP-LF during tilt-up (%) | 48.23±17.31 | 45.61±14.68 | 41.94±13.07 | 59.17±14.02 | 57.83±13.58 | 46.66±14.78 |
| BRS at rest (ms/mmHg) | 5.39±4.02 | 6.35±4.10 | 4.19±4.07 | 4.51±2.43 | 5.12±4.14 | 6.23±4.24 |
| BRS during deep breathing (ms/mmHg) | 6.14±5.23 | 5.70±4.48 | 3.60±2.22 | 4.06±2.33 | 5.10±5.14 | 7.17±4.87 |
| BRS during tilt-up (ms/mmHg) | 3.26±2.45 | 3.66±2.18 | 3.18±3.84 | 2.42±1.53 | 2.18±1.29 | 3.74±4.03 |

BRS=baroreflex sensitivity, HF=high frequency, LF=low frequency, OH=orthostatic hypotension, RRI=RR interval, SBP=systolic blood pressure; OFF-OFF=both off STN-DBS and off dopaminergic medication, ON-OFF=on STN-DBS and off dopaminergic medication, ON-ON=both on STN-DBS and on levodopa, PD=Parkinson's disease. Data are presented as the mean ± SD.
